# Night work and risk of ischaemic heart disease and anti-hypertensive drug use: a cohort study of 145 861 Danish employees

**DOI:** 10.1093/eurpub/ckz189

**Published:** 2019-11-13

**Authors:** Ann D Larsen, Reiner Rugulies, Johnni Hansen, Henrik A Kolstad, Åse Marie Hansen, Harald Hannerz, Anne Helene Garde

**Affiliations:** 1 The National Research Centre for the Working Environment, Copenhagen, Denmark; 2 Department of Public Health, University of Copenhagen, Copenhagen, Denmark; 3 Department of Psychology, University of Copenhagen, Copenhagen, Denmark; 4 Danish Cancer Society Research Center, Copenhagen, Denmark; 5 Department of Occupational Medicine, Danish Ramazzini Centre, University of Aarhus, Aarhus, Denmark

## Abstract

**Background:**

Ischaemic heart disease (IHD) and hypertension are leading causes of mortality and night work has been suspected as a risk factor. Meta-analyses and previous studies are often limited by power and various definitions of exposure and outcomes. This study aimed to investigate if night work increases the risk of IHD or anti-hypertensive drug usage in a large cohort of Danish employees.

**Methods:**

Individual participant data on night work were drawn from the Danish Labour Force Survey (1999–2013). We included 145 861 participants (53% men) 21–59 years of age working 32 h or more per week. Participants with diagnosis or drug use in the year prior to baseline were excluded. Data on outcomes were obtained from nationwide health registers. Using Poisson regression we analyzed incidence rates of the outcomes as functions of night work adjusted for relevant covariates.

**Results:**

We observed 3635 cases of IHD and 20 648 cases used anti-hypertensive drugs. When examining main effects the association of night work with drug use was estimated at rate ratio (RR): 1.05 (95% CI: 1.01–1.09). A sensitivity analysis suggested a dose-response association. The association of night work with IHD was estimated at RR: 1.08 (95% CI: 0.98–1.19). Overall likelihood ratio test showed no statistically significant associations between night work and IHD or drug use when including interactions with sex and socioeconomic status.

**Conclusions:**

Night work was associated with an increased risk of anti-hypertensive drug use. Small estimates suggested a dose-response association. No statistically significant association between night work and IHD were found.

## Introduction

Ischaemic heart disease (IHD) and hypertension are leading causes of mortality. IHD is along with stroke estimated to cause more than 15 million deaths worldwide yearly.^1^ Known risk factors for IHD include family history, diabetes, adverse lifestyle factors and hypertension^2^^,^^3^ but also occupational exposures as night work.^4^^,^^5^ Data from the sixth European Working Conditions Survey from 2015 showed that 19% of the European employees reported working nights at least once a month.^6^

A suggested mechanism linking night work to IHD and hypertension is the disruption of the normal sleep–wake cycle leading to shorter sleep and fatigue.^7^^,^^8^ Insufficient sleep can cause adverse immunological and metabolic changes^9^ and increases the risk of hypertension, diabetes, cardiovascular disease and all-cause mortality.^10–13^ Further, shifts in circadian rhythms have been associated with irregular eating patterns and unhealthy food choices.^14^^,^^15^ An association between night work, IHD and hypertension may also be mediated by psychosocial work stress.^16^^,^^17^

Shift work has been studied in relation to hypertension and IHD, but data on night work is very limited.^18^ In regards to IHD reviews or meta-analyses conclusions were either ‘limited epidemiological evidence’ for causal effect with IHD^19^ or positive association with cardiovascular disease^4^ or more specific: myocardial infarction and ischaemic stroke.^5^ A recent analysis found in the prospective Nurses’ Health Studies that increasing years of rotating night shift work were associated with risk of cardiovascular disease.^20^ This high-quality study was, however, limited by including only female nurses working nights before 1989 in the USA.

In general, the statistical power in the individual studies has often been too low to dismiss or confirm any associations. Instead meta-analyses are used often at the expense that different definitions for exposure and outcomes are pooled. Based on the existing literature there is still a need for large, well-powered studies on the role of night work in the aetiology of IHD and hypertension.

The aim of this study is to investigate the prospective association of night work and IHD or anti-hypertensive drug use in a large, well-powered cohort based on a random sample of the workforce in Denmark. Sex and socioeconomic status (SES) may influence the association, why these are included as interactions terms.

## Methods

Detailed information on data and methods is described in the study protocol that was published before data analysis commenced.^21^

The study was based on representative data from the Danish Labour Force Survey (DLFS), the Danish contribution to the European Labour Force Survey. The DLFS has been conducted as structured telephone interviews by Statistics Denmark since 1994 and each participant could participate was invited for interview four times during 18 months.^22^ Based on the participants’ unique personal identification numbers data from the surveys in the year 1999–2013 was linked to register data.

### Study subjects

We included individuals from DLFS in the time period 1999–2013 for analysis on IHD and 2000–13 for analysis on anti-hypertensive drug use. Participants were 21–59 years of age and worked 32 h or more per week to ensure a homogeneous group and not including early retirements. Those working more than 100 h per week (0.15%) were considered outliers and excluded. Participants were excluded if data on exposure or outcomes were missing. We excluded participants who had been diagnosed with IHD or redeemed prescription for anti-hypertensive drugs in the year prior to baseline interview to limit the likelihood of inclusion of prevalent cases. In total, 145 861 participants were included in the study of IHD and 125 367 participants were included in the study anti-hypertensive drugs. The flow charts are depicted in Supplementary figures S1 and S2.

### Night work

Exposure to night work was based on the participants’ first DLFS interview. There were minor changes in the wording of the question in 2001 and in the response categories in 2006. ‘Before 2001, the participants were simply asked whether they worked at night, but from 2001 onward the question has been whether they worked at night during the last 4 weeks. Until 2006 the response categories were “yes, regularly,” “yes, occasionally” and “no, never”. From 2007 onward the response categories were expanded to “yes, regularly” (i.e. more than half of the working days in the last 4 weeks), “yes occasionally” (i.e. at least once within the last 4 weeks, but less than half of the working days), and “no, not within the last 4 weeks.” Participants who responded with either “yes, regularly” or “yes, occasionally” to the question about night-time work will be defined as being exposed and those who responded with “no…” will be defined as being unexposed to night-time work.’^23^

### Diagnosis of IHD or usage of anti-hypertensive drugs

Information on IHD was obtained from the Danish National Patient Register (DNPR) which included data from all public and private hospitals, outpatients and emergency wards.^24^ Death due to IHD was obtained from the Danish Register of Causes of Death (DRCD).^25^ Usage of anti-hypertensive drugs was obtained from the National Prescription Register (NPR).^26^

From the DNPR and DRCD the ICD-10 codes were: I20 angina pectoris, I21 acute myocardial infarction, I22 subsequent myocardial infarction, I23 certain current complications following acute myocardial infarction, I24 other acute IHDs, I25 chronic IHD. From NPR the ATC-codes were: C02 anti-hypertensive, C03 diuretics, C07 alpha- and beta-blockers, C08 calcium channel blockers and C09 ACE-inhibitors and angiotensin-II antagonists.

### Potential confounders

Time passed since start of follow-up (0–4; 5–9; and ≥10 years) and calendar time (2000–04; 2005–09; and 2010–14) were included as dynamic (time-varying) variables. From DLFS we included information on usual working hours (32–40; 41–48; >48 h a week), defined as the sum of the hours usually worked in primary and secondary jobs. Information on usual working hours was obtained from the baseline interview. Information on sex and age was retrieved from the Central Person Register.^27^ Age was included as a time-varying variable in 10-year classes. From the Employment Classification Module by Statistics Denmark^28^ we included employment in health care (yes vs. no) as working in health care has been associated with referral and prescription bias.^29^ SES was coded in accordance with the three class version of the European Socio-Economic Classification (low, medium, high and unknown).^30^

### Statistical models and test

The participants were followed up, separately, for (i) a first occurrence of a redeemed prescription for antihypertensive drugs and (ii) death due to IHD or a first occurrence of (hospital treatment due to) IHD. The follow-up started at the end of the calendar year of their baseline interview. The follow-up ended when the participant became a case, emigrated or the study period ended (31 December 2014), whichever came first. Poisson regression was used to analyze incidence rates of IHD and anti-hypertensive drug use as a function of night work. ‘Likelihood ratio tests were used to test for main effects as well as for interaction effects between night work and sex or SES. Nested hypothesis testing was used to adjust for multiple comparisons’.^21^

### Sensitivity analyses

We conducted four sensitivity analyses:

- Questions on night work were revised in 2001 and 2007 why we stratified the analysis by calendar period of interview (1999–2000, 2001–06 and 2007–13).
- To test the likelihood of including prevalent cases we excluded all participants who were diagnosed with IHD during a period of 5 years before baseline (opposed to 1 year in the main analysis).
- The probability of awareness of symptoms and the subsequent inclination to seek healthcare may depend on working time arrangements. We therefore performed an analysis which included only diagnosis of acute myocardial infarction (ICD-10: I21) as a more hard endpoint.
- In the primary analysis we used a dichotomised exposure variable for night work (‘Yes’ vs. ‘No’). In this sensitivity analysis, we estimated the rate ratios (RRs) for three ‘Yes, occasionally’ and ‘Yes, regularly’ vs. ‘No’.

## Results

There were 3635 cases of IHD during 1 126 767 person years at risk (PY) corresponding to 32.5 per 10 000 PY and 20 648 cases of anti-hypertensive drug use during 834 551 PY (248.8 per 10 000 PY) from 1999 to 2013. Table 1 presents the descriptive data from both populations (IHD and drug use). Around 13% reported working nights. In the three oldest age groups from 30 to 59 years, the population was equally distributed close to 30%. Only the youngest group was smaller. In the group of night workers the age distribution was similar to the overall population, however more were men (66 vs. 53% in the full population), more came from the low SES group (48 vs. 41%), more were employed in the health care industry (13 vs. 6%), a lower number worked 32–40 h per week (75 vs. 84%) and a higher number worked more than 48 h per week (13 vs. 6%). The distributions were similar in the study population of anti-hypertensive drug usage.

**Table 1:** Participant characteristics in regards to age, sex, SES, employment in healthcare and usual working hours

			IHD				Anti-hypertensive drug usage					
			Night Work				Night work					
	Total		Yes		No		Total		Yes		No	
	*n*	%	*n*	%	*n*	%	*n*	%	*n*	%	*n*	%
	145 861	–	18 658	12.8	127 203	87.2	125 367	–	16 070	12.8	109 297	87.2
Age (years)												
21–29	22 379	15.3	2897	15.5	19 482	15.3	20 515	16.4	2681	16.7	17 834	16.3
30–39	39 721	27.2	5514	29.6	34 207	26.9	36 405	29.0	4947	30.8	31 458	28.8
40–49	42 968	29.5	5724	30.7	37 244	29.3	37 527	29.9	4984	31.0	32 543	29.8
50–59	40 793	28.0	4523	24.2	36 270	28.5	30 920	24.7	3458	21.5	27 462	25.1
Sex												
Men	77 278	53.0	12 261	65.7	65 017	51.1	66 874	53.3	10 617	66.1	56 257	51.5
Women	68583	47.0	6397	34.3	62 186	48.9	58 493	46.7	5453	33.9	53 040	48.5
SES												
High	43 755	30.0	5819	31.2	37 936	29.8	36 673	29.3	5066	31.5	31 607	28.9
Medium	27 644	19.0	1502	8.1	26 142	20.6	23 463	18.7	1287	8.0	22 176	20.3
Low	60 201	41.3	8971	48.1	51 230	40.3	50 765	40.5	7593	47.2	43 172	39.5
Unknown	14261	9.8	2366	12.7	11 895	9.4	12 878	10.3	2124	13.2	10 754	9.8
Employment in health care												
Yes	9009	6.2	2596	13.9	6413	5.0	7745	6.2	2227	13.9	5518	5.0
No	136 852	93.8	16 062	86.1	120 790	95.0	117 622	93.8	13 843	86.1	103 779	95.0
Usual working hours												
32–40/week	122 718	84.1	14 034	75.2	108 684	85.4	104 816	83.6	11 930	74.2	92 886	85.0
41–48/week	14498	9.9	2067	11.1	12 431	9.8	12 978	10.4	1910	11.9	11 068	10.1
>48/week	8645	5.9	2557	13.7	6088	4.8	7573	6.0	2230	13.9	5343	4.9

Table 2 presents the results of the analyses of incident IHD as a function of night work and stratification on sex and SES. All analysis were adjusted for age, time since follow-up, calendar period, employment in health care industry and weekly working hours as well as sex or SES, respectively. In all analyses the group of night workers had higher RR of IHD compared with non-night workers (range for RR: 1.03–1.22) but as indicated by the *P*-values, no statistically significant results were found. When examining night work in a model without interaction effects we observed a RR of 1.08 (95% CI: 0.98–1.19). In the model including interaction terms between night work and sex and SES, we did not find any significant associations.

**Table 2:** RR with 95% CI for incident IHD, as a function of night work among Danish employees 2000–14, with and without stratification by sex and SES, respectively, adjusted for calendar time, time passed since start of follow-up, job in health care sector, age, sex, SES and weekly working hours

Sub-group	Night work	Persons	Person years	Cases	RR	95% CI	*P*-value
All workers	Yes	18 658	147 349	534	1.08	0.98–1.19	Main effect *P* = 0.116
	No	127 203	979 420	3101	1.00		
Male workers	Yes	12 261	96 629	429	1.07	0.96–1.18	Interaction with sex *P* = 0.772
	No	65 017	495 755	2148	1.00		
Female workers	Yes	6397	50 720	105	1.13	0.92–1.39	
	No	62 186	483 665	953	1.00		
Workers with a high SES	Yes	5819	42 478	122	1.15	0.94–1.39	Interactions with SES *P* = 0.757
	No	37 936	266 974	697	1.00		
Workers with a medium SES	Yes	1502	12 150	45	1.22	0.90–1.65	
	No	26 142	208 489	557	1.00		
Workers with a low SES	Yes	8971	74 533	310	1.04	0.92–1.18	
	No	51 230	415 101	1579	1.00		
Workers with unknown SES	Yes	2366	18 188	57	1.03	0.77–1.37	
	No	11 895	88 856	268	1.00		

Table 3 presents the results of the analyses of incident use of anti-hypertensive drugs as a function of night work and further stratified analyses on sex and SES. All analyses were adjusted for age, time passed since start of follow-up, calendar period, employment in health care industry, weekly working hours as well as sex or SES, respectively. When stratifying on sex and SES, the night workers in all strata had higher RRs of incident drug usage compared with non-night workers (RR range: 1.03–1.09), but this was not statistically significant. When examining night work as main effect (in a model without interaction effects), we observed a RR of 1.05 (95% CI: 1.01–1.09) for the association between night work and anti-hypertensive drug usage (*P* = 0.025). In the model including interaction terms between night work and sex and SES, we did not find any significant associations.

**Table 3:** RR with 95% CI for incident use of anti-hypertensive drugs, as a function of night work among Danish employees 2001–14, with and without stratification by sex and SES, respectively, adjusted for calendar time, time passed since start of follow-up, job in health care sector, age, sex, SES and weekly working hours

Sub-group	Night work	Persons	Person years	Cases	RR	95% CI	*P*-value
All workers	Yes	16 070	108 643	2720	1.05	1.01–1.09	Main effect *P* = 0.025
	No	109 297	725 908	17 928	1.00		
Male workers	Yes	10 617	72 472	1755	1.06	1.00–1.11	Interaction with sex *P* = 0.692
	No	56 257	376 827	8736	1.00		
Female workers	Yes	5453	36 171	965	1.04	0.97–1.11	
	No	53 040	349 081	9192	1.00		
Workers with a high SES	Yes	5066	31 607	698	1.06	0.97–1.15	Interaction with SES *P* = 0.315
	No	33 195	203 679	4325	1.00		
Workers with a medium SES	Yes	1287	8894	223	1.03	0.90–1.18	
	No	22 176	152 541	3798	1.00		
Workers with a low SES	Yes	7593	53 453	1452	1.04	0.98–1.10	
	No	43 172	297 180	8047	1.00		
Workers with unknown SES	Yes	2124	14 689	347	1.09	0.97–1.23	
	No	10 754	72 507	1758	1.00		

The first sensitivity analysis stratified by calendar year of interview showed estimates between 1.02 and 1.12 (all statistically non-significant), suggesting that changes in the data collection routines that took place in 2001 and 2007 did not explain any results (table 4).

**Table 4:** RR with 95% CI for incident IHD as a function of night work among Danish employees, stratified by calendar year of interview, adjusted for calendar time, time passed since start of follow-up, job in health care sector, age, sex, SES and weekly working hours

Calendar year of interview	Night work	Persons	Person years	Cases	RR	95% CI
1999–2000	Yes	3522	48 418	173	1.02	0.86–1.20
	No	21 026	291 440	956	1.00	
2001–06	Yes	5943	60 079	230	1.12	0.97–1.30
	No	37 788	384 889	1231	1.00	
2007–13	Yes	9193	38 851	131	1.08	0.90–1.31
	No	68 389	303 090	914	1.00	

The second sensitivity analysis excluding participants who was diagnosed with IHD during a 5-year period prior to baseline, showed a RR for IHD among workers with vs. without night work showed estimates close to those presented in the main analysis (RR: 1.06, 95% CI: 0.96–1.17 vs. RR: 1.08, 95% CI: 0.98–1.19).

The third sensitivity analysis which included 221 cases of myocardial infarction (149.138 PY), showed a RR for myocardial infarction among workers with vs. without night-time work was 1.16 (95% CI: 1.00–1.34).

In the fourth sensitivity analysis where we divided night work into regularly (7677 persons) and occasionally (8393 persons) vs. no night work (109 297 persons), the RR for anti-hypertensive drug use was highest among those having regularly night work (RR: 1.08, 99% CI: 1.02–1.14), followed by those having night work occasionally (RR: 1.02, 99% CI: 0.96–1.08), suggesting a dose–response association.

## Discussion

When examining night work as main effect we observed a modestly increased risk for anti-hypertensive drug use [RR: 1.05 (95% CI: 1.01–1.09)], but not for IHD. We did not find any associations when including interaction terms with night. Sensitivity tests showed a higher rate of myocardial infarction among night workers when compared with non-night workers and further suggesting dose–response association between night work and anti-hypertensive drug use.

The association between night work and use of anti-hypertensive drugs is not fully supported by literature, as a systematic review and meta-analysis on shift and night work and the association with hypertension found shift work to be associated with hypertension (pooled HR 1.31, 95% CI: 1.07–1.60), but not night work.^18^ It should be noted that the data regarding night work and hypertension were very limited. Previous studies found an association between night or shift work and cardiovascular diseases e.g. Torquati et al.^31^ found in their systematic review and meta-analysis a pooled effect size of 1.17 (95% CI: 1.09–1.25) for any cardiovascular events when exposed to shift work. This was supported by the study of Vetter et al.,^20^ who found more than 5 years of rotating night shifts to be associated with higher risk of cardiovascular disease among female nurses [HR(5–9 years) = 1.21, 95% CI: 1.11–1.33, HR(≥10 years) = 1.36, 95% CI: 1.27–1.46] compared with no night work.^20^ This study cannot confirm these findings. It should, however, be noted that differences due to exposure classifications cannot be ruled out as previous studies often use shift work and not night work as exposure. The increased risk of myocardial infarction found in the sensitivity analyses is supported by previous findings presented in a large, systematic review and meta-analysis by Vyas etal.^5^ where shift work was associated with myocardial infarction (pooled RR: 1.23, 95% CI: 1.15–1.31). Since we observed an association between night work and anti-hypertension drug usage but not IHD, it may be speculated if there are different mechanisms potentially linking night work, hypertension and IHD.

### Strengths and limitations

The study has several strengths. We used a prospective study design with a large sample size of 145 861/125 367 persons (IHD/drug usage). The external validity is strengthened by the fact that the DLFS is based on a randomly selected group of the Danish workforce and not on specific industries. Further, both outcomes were based on diagnoses by a physician and not self-report. Linkage to the Danish health registries made loss to follow-up marginal. Further, cardiovascular diagnoses in the Danish National Patient Registry have been validated in 2016 with high positive predictive value.^32^ Due to the large size of the study population in this we have sufficient statistical power to perform analysis stratified by sex, age and SES and thereby provide new insight to such possible differences.

Some limitations need to be addressed. Night work was assessed using a single question without definitions of night work. This crude exposure measurement could cause misclassification bias. However, previous studies have relied on similar information levels^33^^,^^34^ and if there is misclassification, it is probably non-differential. Information on night work was based on self-reports. A recent study from Finland comparing self-reported and register-based information on working time including night work showed however, non-differential exposure misclassification when using self-reported working hours, which would led to an underestimation of the association of working hours and health endpoints.^35^ In our study night work was assessed only once at baseline for a period of the last 4 weeks. It is likely that some workers had different working time arrangements before or after this 4-week period, again leading to exposure misclassification and, assuming that the misclassification was non-differential, to an underestimation of the association with health.

It should also be noted that the prevalence of night work, when estimated by questionnaires is highly dependent on how the questions and response categories are framed. According to a weighted estimate DLFS data, 11% of all employees in Denmark had night-time work in 2012.^36^ In contrast, the Danish National Research Centre for the working environment who uses the question ‘at what time of the day do you usually work in your main job?’ with night-time work defined by the response categories ‘Fixed night work (predominately between 24.00 and 05.00)’ and ‘Shift work, including night time work’, estimates the prevalence of night time work among employees in Denmark 2012 at 7.3%.^37^ Number of years exposed to night work are not included in the DLFS, we were therefore not able to test to if increasing years of night work could lead to IHD, as reported by Vetter et al.^20^ Further, we were not able to test how years without night work affected the risk of IHD.

We did not have information on all relevant risk factors of IHD e.g. smoking and BMI. We therefore included SES as a crude proxy for relevant health-behaviour related factors, as studies have shown low SES to be associated with health behaviours such as smoking and BMI.^38^ Yet, the effects of health behaviour cannot be ruled out and given the small size of the effect estimates as well as the possibility of prescription bias^29^ conclusions should be made cautiously.

We excluded prevalent cases i.e. participants with IHD or drug usage in the year prior to baseline. One year was chosen in order to minimize loss of participants who could not be followed back in the registers. Sensitivity analysis showed similar estimates for IHD with exclusion of 1 year RR: 1.08 (95% CI: 0.98–1.19) and 5 years (sensitivity test): 1.06 (95% CI: 0.96–1.17). We therefore assume that limiting the exclusion of prevalent cases to the year preceding the baseline assessment has not introduced bias towards unity into our analyses.

Further, we need to underline that the hierarchy of analytical testing (described in the statistics section) were changed after the publication of the protocol^21^ as the arguments for the testing hierarchy only applied to long working hours which the protocol also covers.

In conclusion, night work was associated with an increased risk of anti-hypertensive drug use. The estimates were small but suggested a dose-response association. We did not find a statistically significant association between night work and incident IHD.

## Funding

The study was funded by the Danish Working Environment Research Fund under grant number 38-2013-09/20130069288. The Research Fund had played no role in planning, execution or interpretation of the study or in the decision of publishing. The study is also carried out within the framework of the WOW project funded by NordForsk, Nordic Programme on Health and Welfare (74809) without further involvement in the project.

*Conflicts of interest*: None declared.

**Key points**

- Based on more than 125 000 participants night work was found to be associated with a modest increased risk of anti-hypertensive drug usage.
- We found no association between night work and incident ischaemic heart disease (IHD).
- This well-powered study provides new knowledge relevant for policy makers of the linkage between a common exposure as night work and leading causes of mortality as IHD and hypertension.

## Supplementary Material

ckz189_Supplementary_Data
