# Supplementary material for: Night work and risk of ischaemic heart disease and anti-hypertensive drug use: a cohort study of 145 861 Danish employees
Source: Eur J Public Health. 2019 Nov 13;30(2):259–64. doi: 10.1093/eurpub/ckz189 (PMC7183362; doi:10.1093/eurpub/ckz189)
Supplement: ckz189_Supplementary_Data [file ckz189_supplementary_data.zip › ckz189-Suppl_Data/ejph-2018-12-om-1101-File003.docx]

Participated in the Danish Labour Force Survey sometime during the time period 1999 – 2013 (N = 357 085)

Excluded for not being 20 – 59 years old at baseline (N = 108 373)

Excluded for not being employed (N = 65 580)

Excluded for working less than 32 hours per week (N = 33 277)

Excluded due to emigration during the calendar year preceding baseline (N = 436)

Excluded for not being found in national registers (N = 11)

Excluded due to missing data on night work (N = 709)

Included in the analysis of IHD

(N = 145 861)

Excluded for working more than 100 hours per week (N = 294)

Excluded due to hospital diagnosed IHD during the calendar year preceding baseline (N = 427)

Excluded due to missing industrial code (N = 2117)

Figure 1. Flowchart, study population for analyses on night work and risk of IHD
