# Supplementary material for: Night work and risk of ischaemic heart disease and anti-hypertensive drug use: a cohort study of 145 861 Danish employees
Source: Eur J Public Health. 2019 Nov 13;30(2):259–64. doi: 10.1093/eurpub/ckz189 (PMC7183362; doi:10.1093/eurpub/ckz189)
Supplement: ckz189_Supplementary_Data [file ckz189_supplementary_data.zip › ckz189-Suppl_Data/ejph-2018-12-om-1101-File004.docx]

Participated in the Danish Labour Force Survey sometime during the time period 2000 – 2013 (N = 341 482)

Excluded for not being 20 – 59 years old at baseline (N = 106 006)

Excluded for not being employed (N = 61 563)

Excluded for working less than 32 hours per week (N = 31 568)

Excluded due to emigration during the calendar year preceding baseline (N = 407)

Excluded for not being found in national registers (N = 10)

Excluded due to missing data on night work (N =661)

Included in the analysis of anti-hypertensive drug usage

(N = 125 367)

Excluded for working more than 100 hours per week (N = 289)

Excluded due to redeemed prescriptions for anti-hypertensive drugs during the calendar year preceding baseline (N = 13 626)

Excluded due to missing industrial code (N = 1985)

Figure 2. Flowchart, study population for analyses on night work and risk of anti-hypertensive drug usage
